# DNA nanochannels

**DOI:** 10.12688/f1000research.10464.1

**Published:** 2017-04-18

**Authors:** Dianming Wang, Yiyang Zhang, Dongsheng Liu

**Affiliations:** 1 Department of Chemistry, Tsinghua University, Beijing, China

**Keywords:** DNA nanotechnology, nanostructures, tile-based nanotube, origami nanochannel, bundle nanochannel

## Abstract

Transmembrane proteins are mostly nanochannels playing a highly important role in metabolism. Understanding their structures and functions is vital for revealing life processes. It is of fundamental interest to develop chemical devices to mimic biological channels. Structural DNA nanotechnology has been proven to be a promising method for the preparation of fine DNA nanochannels as a result of the excellent properties of DNA molecules. This review presents the development history and current situation of three different types of DNA nanochannel: tile-based nanotube, DNA origami nanochannel, and DNA bundle nanochannel.

## Introduction

DNA has been proven to be an excellent structural molecule besides being used for genetic information storage and transfer because the specificity of the base pairing allows target structures to be encoded in the base sequences of component oligonucleotides. After more than 30 years of development since Nadrian Seeman proposed that DNA could be used to construct junction structures in 1982 ^1^, molecular self-assembly by DNA has enabled the construction of arbitrary objects with nanometer- to micrometer-scale dimensions (for example, crystals, patterns, bricks, boxes, and curved shapes) ^2– 4^. These nanostructures either perform functions independently or simulate other biomolecules; among them, the DNA nanochannel is one of the finest examples that could mimic the biological nanochannels in both structure and function. Biological nanochannels are widely distributed in nature; they play important roles in various biological processes, regulating the transport of materials and signals through biomembranes ^5, 6^. An accurate understanding of the structures and functions of the nanochannels would help us unveil the life processes and has important guiding significance for drug design, disease diagnosis, and manufacturing of bio-inspired devices. In this review, we will briefly introduce the development history and current situation of DNA nanochannels in three classifications: tile-based nanotube, DNA origami nanochannel, and DNA bundle nanochannel.

## Tile-based nanotube

**Figure 1.**
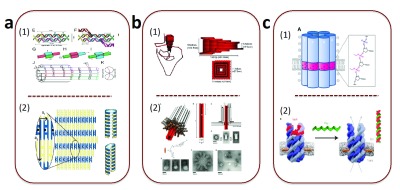
DNA nanochannels. ( **a**) Tile-based nanotube. ( **b**) DNA origami nanochannel. ( **c**) DNA bundle nanochannel.

The approach of DNA tile-based structures was to use DNA to construct relatively simple tiles such as the double crossover (DX) ^7^ or triple crossover ^8^. Then, these preformed tiles could assemble into periodic lattices through DNA hybridization. The DNA tiles could be assembled into a variety of shapes; moreover, they could be used to implement algorithmic self-assembly, making them a platform for DNA computing ^9^. In 2004, two articles focusing on the DNA tile-based nanotube were published in the *Journal of the American Chemical Society* in the same issue ^10, 11^ ( Figure 1a). Both of these works take advantage of DX to construct the nanotubes. Owing to the periodic array of DX, the nanotubes were borderless in theory. In their experiments, both sets of investigators observed micrometer-scale nanotubes under atomic force microscope. In 2008, Yin *et al.* used a single-strand tile strategy to construct the DNA nanotube ^12^. The tile in this strategy, unlike the previous method, was only the DNA single strand. Another way to build the DNA nanotube was to use small circular DNA molecules as tiles and then introduce assisted staple strands to connect the circular DNA by forming crossover ^13^. Owing to the designability and biological compatibility, the tile-based nanotubes were widely used as the one-dimensional templates ^14^ and nanowires ^15^. Nevertheless, it was difficult to control their shape and size because of the periodic array of the tile, which made it hard to mimic the biological nanochannel. However, the DNA origami provided a promising way to solve this problem.

## DNA origami nanochannel

The concept of DNA origami was first proposed by Rothemund in 2006; it was assembled by folding a long single-stranded DNA into designed structures with the aid of multiple short staple strands ^16^. It has been widely used as a bottom-up approach for the assembly of versatile nanostructures. In 2012, Keyser *et al.* constructed a funnel-shaped three-dimensional DNA origami nanochannel, which could be inserted into the silicon nitride nanopores ^17^. The designed shape enabled the structure to fit into the solid pore size ( Figure 1b1). When the DNA origami was trapped into the solid-state nanopore, it reduced the flow of ions and therefore a characteristic drop in conductance was observed. It had been proven that hybrid DNA origami-solid-state nanopores could be used as the resistive-pulse sensor for λ-DNA. The following year, Simmel *et al.* published a syringe-shaped DNA origami nanochannel ^18^. This nanochannel could penetrate and span a lipid membrane by modifying 26 cholesterols around the channel, making the structure more closely mimic the action of biological protein nanopores ( Figure 1b2). It has also been proven that this nanochannel possessed the property of electrical conductivity by single-channel electrophysiological experiments using an integrated chip-based setup. In addition to allowing small ions to pass through, the DNA origami nanochannel could modulate molecule transport. In 2016, Krishnan *et al.* reported a bigger T-shaped DNA origami nanochannel incorporated into a membrane ^19^. This nanochannel was composed of a double-layered top plate and a 27 nm-long stem attached to the center of the plate. The stem was formed by 12 helices arranged in a square with a side length of 4.2 nm. The plate provides a large area for membrane interactions. Furthermore, Krishnan *et al.* had investigated an alternative strategy for inserting and anchoring DNA nanochannels into lipid membranes, which uses biotinylated lipid molecules. The specific interaction of biotinylated lipid-streptavidin and biotinylated anchor strands on DNA origami provided a different approach for interaction and methods to avoid the aggregation of DNA nanochannels. Coincidentally, Keyser *et al.* created a larger funnel-shaped DNA origami nanochannel the same year ^20^. The cross-section of the DNA nanochannel was 6 nm. Nineteen cholesterol anchors were used to facilitate insertion into a lipid membrane. Fan *et al.* constructed a DNA origami nanochannel with a diameter of 22 nm as the addressable bioreactor ^21^. They incorporated a couple of cascade enzymes into the nanochannel lumen. Compared with the free state in the solution, the nanochannel could notably enhance the cascade reaction efficiency. In 2015, Kostiainen *et al.* reported a modularity nanochannel based on the DNA origami strategy ^22^. They could controllably combine separate DNA nanochannel units equipped with a couple of cascade enzymes and demonstrate an efficient enzyme cascade reaction inside the nanochannel. The following year, Liu *et al.* reported a smart DNA nanochannel with a shutter at the end, which could be reversibly switched open and closed, using a DNA chain exchange reaction ^23^. They could regulate the transport of molecules into the nanochannel in nanoscale by controlling the state of the shutter.

## DNA bundle nanochannel

Unlike DNA origami, which requires a long chain template, the DNA bundle nanochannel was very simple; it was formed by only a few oligonucleotides. This kind of nanochannel was composed of a bundle of six interconnected DNA duplexes, with an aperture of about 2 nm. The DNA bundle channel was inserted into the lipid membrane by decoration of the outer layer with hydrophobic residues ^24^. Howorka *et al.* used the hydrophobic ethyl-phosphorothioate (ethyl-PPT) DNA backbone instead of the conventional phosphate ** DNA backbone to construct the nanochannel ^25^. This way, the barrel featured a hydrophobic belt composed of 72 ethyl groups, which endowed the nanochannel with the ability of spanning the lipid membrane ( Figure 1c1). The authors proved that this nanochannel could be inserted into lipid bilayers and support a stable ionic current by single-channel current analysis. Furthermore, this DNA bundle nanochannel could interact with cellular membranes and exert a cytotoxic effect ^26^. Besides the capping of ethyl around the phosphate backbone, nucleoside modification was reported as a possible way to enable the insertion of the DNA nanochannels into lipid membrane. In particular, tetraphenylporphyrin (TPP) tags were introduced to the similar six-duplex bundle nanochannel, which could anchor the channel into the lipid bilayer, via the Sonogashira reaction between an acetylene-TPP derivative and a deoxyuridine nucleoside ^27^. Because the hydrophobicity of TPP is much stronger than that of the ethyl groups, only two TPP molecules were enough to insert the channel into the lipid membrane. In addition, the TPP possessed fluorescence emission, allowing the direct visualization of the anchored nanochannel once incubated with giant unilamellar vesicles. In 2016, Howorka *et al.* reported a gating nanochannel based on a similar six-bundle nanochannel ^28^. A lock DNA could hybridize with the channel to close it, and additional key DNA would replace the lock DNA from the channel and as a result the channel opened ( Figure 1c2). Once this gating nanochannel is inserted into the giant unilamellar vesicles with the assistance of three cholesterols, the switch of the nanochannel could regulate the release behavior of small molecules in the vesicles. Besides reporting the six-DNA bundle nanochannel, Keyser *et al.* reported a nanochannel composed of only four DNA bundles, which were arranged on a square lattice ^29^. In this arrangement, the diameter of the central channel in the middle of the four helices was only about 0.8 nm. Similarly, two cholesterols here were used to assist the channel to be inserted into the lipid membrane.

## Conclusions and prospect

In this review, we have outlined the development history and current situation of the nanochannel based on the structural DNA nanotechnology. So far, three different types of DNA nanochannel have been reported: the tile-based nanotube, the DNA origami nanochannel, and the DNA bundle nanochannel. It has been proven that the DNA nanochannel was promising in the area of bio-mimic channel and molecule transport regulation of nanoscale. Furthermore, the DNA nanochannel could tune membrane fluidity and trigger a cytotoxic effect. However, this is an emerging field that in many respects is still in its infancy: one of the major challenges with the DNA nanochannels is to improve their stability for the purpose of avoiding the ionic leakage caused by fluctuations of the nanochannel in the membrane. In addition, at present, there is only one kind of responsiveness for the DNA nanochannel, and how to introduce more responsiveness into the nanochannel to better mimic the native protein channel is still a big challenge. We believe that more and more intelligent DNA nanochannels, inspired by the DNA-modified solid-state nanochannels ^30, 31^, which possessed different kinds of responsiveness, will be developed in the future.
